# Obituary. Rev. Asher Wright

**Published:** 1875-04

**Authors:** 


### OBITUARY.

Died, April 13, 1875, at his residence, Versailles, Cattaraugus Co., N. Y., Rev. Asher Wright. To those who were unacquainted with Rev. Mr. Wright and his years of faithful self-sacrifice and toil, this brief notice would have but little import; to others it will bring the intelligence that a respected and revered friend has gone to his rest after forty years of labor among the Indians as Pastor and Physician. Mr. Wright was born in September, 1803, and received, we believe, a collegiate education. After preparing himself for the ministry, he resolved to devote his life to the work of christianizing the Indians. He came to the Buffalo Creek Reservation in 1830, and commenced his labors among the remnants of the tribes forming the Six Nations. When, in 1845, the Indians removed to the Cattaraugus Reservation he went with them, and has since remained there a beloved and honored counselor and friend. Mr. Wright not only ministered to the spiritual wants of his charge, but he also made himself familiar with the science of medicine, and was thus enabled to minister to their physical infirmities. We do not know that he ever took a degree in medicine, but we have ever known him as a faithful, conscientious and intelligent physician—one who was ever anxious to do the best that he could for the people of his charge.

Among the Indians he was universally respected and loved, and they will long mourn his departure. We can pronounce no higher eulogy than to say that in all respects, as Pastor, Physician or Friend, he was a thoroughly good man.

